# Draft Genome Sequence of Halomonas urumqiensis BZ-SZ-XJ27^T^, an Aerobic Halophilic Bacterium Isolated from a Salt Lake

**DOI:** 10.1128/MRA.00877-18

**Published:** 2018-07-26

**Authors:** Ziya Liao, Dacheng Qiu, Haisheng Wang, Yanchun Yan, Jun Li, Baisuo Zhao

**Affiliations:** a Graduate School, Chinese Academy of Agricultural Sciences, Beijing, People’s Republic of China; b Institute of Agricultural Resources and Regional Planning, Chinese Academy of Agricultural Sciences, Beijing, People’s Republic of China; University of Delaware

## Abstract

The aerobic halophilic bacterium Halomonas urumqiensis BZ-SZ-XJ27^T^, growing optimally at 1.42 M Na^+^, with a range of 0.22 to 4.32 M Na^+^, was isolated from a salt lake in the Xinjiang Uyghur Autonomous Region of China. Here, we report the draft genome sequence of strain BZ-SZ-XJ27^T^, which consists of approximately 3.97 Mb and contains 3,588 predicted genes. Some of the genes that maintain intracellular osmotic balance were identified, offering valuable insights into specific adaptations to the hypersaline environment.

## Abstract (precis)

The aerobic halophilic bacterium Halomonas urumqiensis BZ-SZ-XJ27^T^, growing optimally at 1.42 M Na^+^, with a range of 0.22 to 4.32 M Na^+^, was isolated from a salt lake in the Xinjiang Uyghur Autonomous Region of China. Here, we report the draft genome sequence of strain BZ-SZ-XJ27^T^, which consists of approximately 3.97 Mb and contains 3,588 predicted genes.

## ANNOUNCEMENT

The moderately halophilic Halomonas urumqiensis BZ-SZ-XJ27^T^ was aerobically isolated from a salt lake in the Xinjiang Uyghur Autonomous Region of China (1). Halomonas urumqiensis BZ-SZ-XJ27^T^, growing at total Na^+^ concentrations of 0.22 to 4.32 M, with optimum growth at 1.42 M, is affiliated with the family Halomonadaceae of the class Gammaproteobacteria and is most closely related to Halomonas korlensis XK1^T^ and Halomonas campaniensis 5AG^T^. To understand the osmotic adaptation mechanism for survival under hypersaline conditions, the draft genome sequencing of strain BZ-SZ-XJ27^T^ was carried out using an Illumina platform.

Total genomic DNA (2 μg) was isolated using the iTop microbial DNA isolation kit (Beijing, China), according to the manufacturer's instructions. An Illumina sequencing library was created using whole-genome shotgun assembly (2), and next-generation sequencing was performed on an Illumina MiSeq sequencer. Sequencing was achieved with a paired-end read length of 2 × 300 bp at approximately 159× coverage. The read was quality trimmed using Quake and BWA and *de novo* assembled into contigs using A5-miseq version 20150522 (3–5). A total of 2,438,804 reads with a total length of 3,978,292 bp were assembled into 20 contigs with a G+C content of 62.6% and an *N*_50_ value of 561,230 bp. Automated gene annotation was obtained using PGAP (www.ncbi.nlm.nih.gov/genome/annotation_prok). Subsequently, the genome file of GenBank format (gb file) was uploaded to IMG-ER (https://img.jgi.doe.gov/cgi-bin/submit/main.cgi) for functional annotation after registering an analysis project identification (ID) in the Gold Database (https://gold.jgi.doe.gov/index). Among the 3,588 genes predicted, 3,524 were potential protein-coding genes (CDS). Also identified were 64 RNAs, including 6 rRNAs (4 5S RNAs, 1 16S RNA, and 1 23S RNA), 55 tRNAs, and 3 other RNAs.

Genome sequence analysis revealed that strain BZ-SZ-XJ27^T^ harbors a number of genes that achieve the maintenance of osmotic balance though the “compatible-solutes strategy” under high-salt conditions, including 1 gene cluster (*ectA*, *ectB*, and *ectC*) for ectoine biosynthesis from aspartate semialdehyde, 2 *betA* genes and 4 *betB* genes for glycine betaine biosynthesis from choline, 3 *glnA* genes for l-glutamine biosynthesis from l-glutamate, the *proV* gene*, proW* gene, and 2 *proX* genes for glycine betaine/proline ABC transporters, 7 genes for glycine betaine/carnitine/choline transporters (BCCT family), and 4 genes for Na^+^/solute symporters (6–9). In addition, the analysis showed 5 genes (3 TrkA type and 2 TrkH type) are responsible for K^+^ uptake systems, implying that strain BZ-SZ-XJ27^T^ may gain isosmotic cytoplasm though K^+^ as an osmolyte via a “salt-in strategy” when coping with a rapid osmotic shock (10). Furthermore, 7 genes of the multisubunit Na^+^/H^+^ antiporter-3 (Mrp antiporter, CPA-3 family) were found to be present (11), and 1 gene of the monovalent cation/H^+^ antiporter-1 (CPA-1 family) (12) and 1 gene of the Na^+^/H^+^ antiporter (NhaD family) that discharges Na^+^ from the cytoplasm also lead to a broad spectrum of moderate salinities (13). In this report, the predicted genes play important roles in maintaining osmotic balance and Na^+^ homeostasis under salinity stress.

### Data availability.

The draft genome sequence of Halomonas urumqiensis BZ-SZ-XJ27^T^ has been deposited at DDBJ/ENA/GenBank under the accession number PYUD00000000.
